# Children gut microbiota exhibits a different composition and metabolic profile after *in vitro* exposure to *Clostridioides difficile* and increases its sporulation

**DOI:** 10.3389/fmicb.2022.1042526

**Published:** 2022-12-09

**Authors:** Sabina Horvat, Aleksander Mahnic, Damjan Makuc, Klemen Pečnik, Janez Plavec, Maja Rupnik

**Affiliations:** ^1^Department of Microbiology, Faculty of Medicine, University of Maribor, Maribor, Slovenia; ^2^Centre for Medical Microbiology, National Laboratory of Health, Environment and Food, Maribor, Slovenia; ^3^Slovenian NMR Centre, National Institute of Chemistry, Ljubljana, Slovenia

**Keywords:** *Clostridioides difficile*, gut microbiota, sporulation, metabolomic, children

## Abstract

*Clostridioides difficile (Clostridium difficile)* infection (CDI) is one of the main public health concerns in adults, while children under 2 years of age are often colonized asymptomatically. In both adults and children, CDI is strongly associated with disturbances in gut microbiota. In this study, an *in-vitro* model of children gut microbiota was challenged with vegetative cells or a conditioned media of six different toxigenic *C. difficile* strains belonging to the ribotypes 027, 078, and 176. In the presence of *C. difficile* or conditioned medium the children gut microbiota diversity decreased and all main phyla (*Bacteroidetes, Firmicutes*, and *Proteobacteria*) were affected. The NMR metabolic spectra divided *C. difficile* exposed children gut microbiota into three clusters. The grouping correlated with nine metabolites (short chain fatty acids, ethanol, phenolic acids and tyramine). All strains were able to grow in the presence of children gut microbiota and showed a high sporulation rate of up to 57%. This high sporulation rate in combination with high asymptomatic carriage in children could contribute to the understanding of the reported role of children in *C. difficile* transmissions.

## Introduction

*Clostridioides (Clostridium) difficile* is a well-established causative agent of gastrointestinal infections associated with antibiotic therapy in humans worldwide (Martin et al., 2016). The symptoms range from asymptomatic colonization to mild diarrhea and more severe manifestations, i.e., pseudomembranous colitis or gut perforation. The outcome of *C. difficile* infection (CDI) depends on the type of the *C. difficile* strain (i.e., toxigenic profile, ribotype) and host factors (i.e., age, immune response; Rupnik et al., 2009; Leffler and Lamont, 2015). While the asymptomatic carriage of *C. difficile* is rare in healthy adults (4–15%, Schnizlein and Young, 2022), the high rate of asymptomatic colonization in children under 2 years of age is well-documented and reaches up to 75% (Collignon et al., 1993; Fallani et al., 2006; Jangi and Lamont, 2010; Rousseau et al., 2011; Azad et al., 2013). The colonization rate is the highest between 6 and 12 months (Tougas et al., 2021). In addition, young children are often colonized by toxigenic strains while remaining asymptomatic (Collignon et al., 1993; Jangi and Lamont, 2010; Adlerberth et al., 2014). Therefore, children have long been considered to be at low risk for symptomatic CDI but the epidemiology of CDI in children has changed over the past decade (Kociolek et al., 2021). Similar to adult infections, severe cases, hypervirulent strains, more community-onset infections without previous antibiotic exposure, and outbreaks have been observed in pediatric populations (Noor and Krilov, 2018; Kociolek et al., 2021).

The interactions between *C. difficile* and the gut microbiota significantly contribute to the development of CDI, but studies on microbiota in children in the context of CDI or carriage are rare (Rousseau et al., 2011; Davis et al., 2016; Drall et al., 2019; Smith et al., 2020). A healthy and fully developed microbiota provides colonization resistance based on several mechanisms ranging from bile salts metabolism to nutrient limitation (Britton and Young, 2012; Theriot and Young, 2015; Collins and Auchtung, 2017; Schnizlein and Young, 2022). However, recent studies show that *C. difficile* can also influence the gut microbiota. It decreases the diversity, changes the composition and specifically affects certain bacterial groups through diverse mechanisms (Horvat et al., 2017; Horvat and Rupnik, 2018; Jenior et al., 2018; Fletcher et al., 2021). These results suggest that *C. difficile* could induce and/or maintain alterations in the gut microbiota composition in order to thrive in the gut, as was previously demonstrated for some other gut pathogens (Lupp et al., 2007; Stecher et al., 2007).

We and others have shown that *C. difficile* could grow *in vivo* and *in vitro* in the presence of a healthy microbiota (Robinson et al., 2014; Horvat and Rupnik, 2018). Moreover, while the growth in the presence of microbiota was generally poorer, sporulation was increased. In our previous study, the growth, sporulation, and level of microbiota disruption differed among microbiota samples from healthy adult individuals and adults with disrupted microbiota (Horvat and Rupnik, 2018). In this study, we aimed to explore the growth and sporulation of *C. difficile* in the presence of fecal microbiota of healthy children aged under 2 years, and to search for possible effects of *C. difficile* vegetative cells and conditioned medium on children microbiota composition and its metabolic profile. Six different toxigenic *C. difficile* strains, belonging to ribotypes 027, 078 and 176 were used. Epidemic ribotypes 027 and 078 are generally linked with higher morbidity and mortality, whereas ribotype 176 emerged recently and is closely related to ribotype 027 (Rupnik et al., 2016; Couturier et al., 2018; Novakova et al., 2020).

## Materials and methods

### *Clostridioides difficile* strain selection and handling

Six different *C. difficile* toxigenic strains were selected from our *C. difficile* strain collection. Strains belonged to PCR ribotypes 027 [strain designations E27 (ZZV09-2033) and 1998 (ZZV14-5907)], 078 [strain designations W43018 (ZZV07-177) and 4,894-4III (ZZV10-2591)] and 176 [strain designations 3,014 (ZZV16-7488) and 1974/12 (ZZV16-7558)]. Strains were collected from different countries between the years 2006 and 2016. Five of the selected strains originated from humans with CDI, while one ribotype 078 strain originated from cattle.

For fecal microbiota cultivation we have prepared a *C. difficile* conditioned medium as described previously (Horvat et al., 2017) with some modifications. Selected strains were incubated anaerobically at 37°C for 48 h in a Wilkins Chalgren Anaerobe Broth (WCAB; Oxoid) to obtain *C. difficile* suspensions with up to 10^10^ colony forming units (CFU)/ml, respectively. After a centrifugation at 10,000 rpm for 5 min, the supernatants were filtered (0.2 μm, cellulose acetate, Sarstedt) to obtain a conditioned medium, which was used for *in vitro* cultivation procedures.

### Preparation of fecal inoculum

A pooled fecal slurry from four children was used in all experiments. An informed consent was obtained from parents. Ethic approval was obtained by Ethic Committee of Medical faculty, University of Maribor. *C. difficile* negative fecal specimens were obtained from healthy children under 2 years of age (female: *n* = 3, male: *n* = 1). Children were from 12 to 22 months old and were healthy, with no history of antibiotic treatment within 3 months before sampling. During sampling all were introduced to solid foods with occasional breastfeeding.

After collection, fecal specimens were stored at 4°C for a maximum of 24 h prior to transport to the laboratory. Upon sample delivery 0.2 g aliquots were prepared and frozen at −80°C until further processing.

All selected specimens were confirmed as *C. difficile* negative with real-time PCR specific for 16S rDNA gene of *C. difficile* using primers and conditions as previously described (Rinttila et al., 2004) and the probe 5′-CCTACCCTGTACACACGGATAAC ATACCG-3′.

Before the *in vitro* experiment, one aliquot of each specimen was thawed and diluted in pre-reduced WCAB growth medium to obtain 10% fecal slurry. Equal amounts of fecal slurries of individual samples were pooled, added to pre-reduced WCAB growth medium (1,100, v:v) and incubated anaerobically at 37°C overnight to obtain fecal inoculum for the *in vitro* cultivation procedures.

The pooled fecal slurry (fecal input sample) was included as a control for the 16S rDNA sequencing to test for changes in microbiota composition after *in vitro* cultivation.

### *In vitro* cultivation of fecal microbiota in a *Clostridioides difficile* conditioned medium

The fecal microbiota was cultured in a *C. difficile* conditioned medium as described previously (Horvat et al., 2017) with minor modifications. The overnight fecal inoculum (50 μl) was added into pre-reduced WCAB conditioned media (4.95 ml) in a 6-well plate (Sarstedt). The control fecal inoculum was grown in a freshly prepared and pre-reduced WCAB growth medium. Each described culture condition was done in triplicate. The cultures were incubated with gentle mixing for 72 h anaerobically at 37°C. After the incubation, the samples (5 ml) were centrifuged at 10,000 rpm for 10 min. Pellets were used for total bacterial DNA extraction and the supernatants were screened for metabolic spectra with NMR spectroscopy (described below).

### *In vitro* co-cultivation of fecal microbiota and *Clostridioides difficile* vegetative cells

For co-cultivation procedures the overnight culture of the fecal inoculum was combined with suspensions of *C. difficile* vegetative cells as described previously (Horvat and Rupnik, 2018). Briefly, overnight *C. difficile* cultures were inoculated into pre-reduced WCAB growth medium (1:500, v:v) and incubated for 17 h to obtain suspensions of *C. difficile* vegetative cells with up to 2 × 10^8^ CFU/ml. Fecal inoculum was prepared as described above. Fecal inoculum (50 μl of overnight culture in WCAB) and *C. difficile* vegetative cells (50 μl) were added to pre-reduced WCAB growth medium (4.9 ml) in a 6-well plate (Sarstedt). Initial concentration of *C. difficile* in co-cultures was approximately 2 × 10^6^ CFU/ml. Cultures were incubated for 3 days under anaerobic conditions and with gently mixing. Each described growth condition was done in triplicate. Monocultures of *C. difficile* or fecal microbiota alone were used as controls. After 72 h of anaerobic incubation at 37°C two aliquots of 100 μl were taken for total viable cell and spore count of *C. difficile* (described below). The remaining 4.8 ml were centrifuged at 10,000 rpm for 10 min. The pellets were used for total bacterial DNA extraction and the supernatants were screened for metabolic spectra with NMR spectroscopy (described below).

### Monitoring *Clostridioides difficile* growth and sporulation

*The C. difficile* total viable cell count was determined as CFUs on chromID *C. difficile* agar plates (bioMerieux) and the *C. difficile* spores were determined as CFUs on chromID *C. difficile* agar plates after a 30-min treatment with 100% ethanol. The frequency of sporulation was defined as the percentage of the ethanol-resistant CFU relative to the total CFU. One-way ANOVA with a Bonferroni correction was used in the statistical analysis to determine statistically significant differences between samples.

### 16S rDNA amplicon sequencing and sequence data analysis

The isolation of total DNA was performed using the QIAamp Fast Stool DNA Mini Kit (Qiagen) after a mechanical disruption (speed 7,000 rpm for 70 s) with the SeptiFast Lyse Kit (Roche) on MagNA Lyser (Roche). The quantification of DNA was performed with the Quant-iT PicoGreen dsDNA Kit (Thermo Fisher Scientific). The amplification of the V3–V4 region of the 16S rRNA gene was carried out using the 341F (5′-CCTACGGGNGGCWGCAG-3′)–805R (5′-GACTACHVGGGTATCTAATCC-3′) set of primers. The amplicons were paired-end sequenced on an Illumina MiSeq platform (2 × 300 cycles). On the same run a template-free sample was included as a negative sequencing control.

The MiSeq output data was analyzed according to the MiSeq standard operating procedure (SOP) for Illumina paired-end reads (Kozich et al., 2013) with the mothur software (v.1.36.1; Schloss et al., 2009). The analysis was done according to following criteria: (i) the sequences were not allowed any ambiguous bases or more than eight homopolymers, (ii) the sequences were aligned against the Silva v132 reference alignment (Quast et al., 2013), (iii) chimeras were found with the UCHIME algorithm (Edgar et al., 2011) and removed, (iv) classification of the sequences was performed using the RDP training set (v.9) with an 80% confidence threshold, (v) the sequences of bacterial origin only were clustered into operational taxonomic units (OTUs) at a 3% dissimilarity level, (vi) OTUs with a relative abundance lower than 0.001% were excluded from further analysis. To characterize the alpha samples diversity, the Shannon index was chosen. Beta diversity was estimated with the AMOVA (Schloss, 2008) and weighted UniFrac measures (Lozupone et al., 2007). MEGA software (v.7.0.26; Kumar et al., 2016) was used for the hierarchical clustering of the samples. The OTUs that differed between treatments were searched with the linear discriminant analysis (LDA) effect size (LEfSe) method (Segata et al., 2011).

The sequence data was deposited on the Metagenomics RAST (MG-RAST) database server^1^ under the project access number mgp90153.^2^

### NMR analysis of extracellular metabolites

The supernatants were filtered (0.2 μm, cellulose acetate, Sarstedt) and transferred into 5 mm FEP Tube Liner for 7” NMR Sample Tube (Wilmad-LabGlass) for NMR spectroscopy analysis. The reference compound 3-(trimethylsilyl) propionic acid sodium salt (TMSPA) was added to each sample. The NMR spectra were acquired on a 600 MHz NMR spectrometer (Agilent Technologies). Spectral preprocessing was done using ACD labs software (ACD/Spec Manager, v.12.01). All NMR spectra were phased, baseline-corrected and referenced to the TMSPA signal. The reference signal TMSPA and the region around the water signal between δ 4.65 and 5.35 ppm were excluded from the data in order not to interfere with the analysis of the metabolites. Each ^1^H NMR spectrum between δ 0.1 and 9.5 ppm was segmented into buckets with 50% looseness on widths of 0.04 ppm using ACD labs intelligent bucketing algorithm. The area under each binned region was integrated and the integrated binned regions were normalized to the ‘constant sum’ equal to 100. 2D NMR spectra were processed and analyzed using VNMRJ (Agilent-Varian) and Sparky (UCSF) software as previously described (Pecnik et al., 2018). Random Forests analysis was performed using MATLAB’s TreeBagger algorithm (MATLAB Statistics and Machine Learning Toolbox, v.R2016b) with 30 bagged classification trees. A MATLAB m-file for the general explanation method was prepared by modification of previously published algorithm (Strumbelj and Kononenko, 2010).

## Results

### An increased *Clostridioides difficile* sporulation in the presence of children fecal microbiota

**Figure 1:**
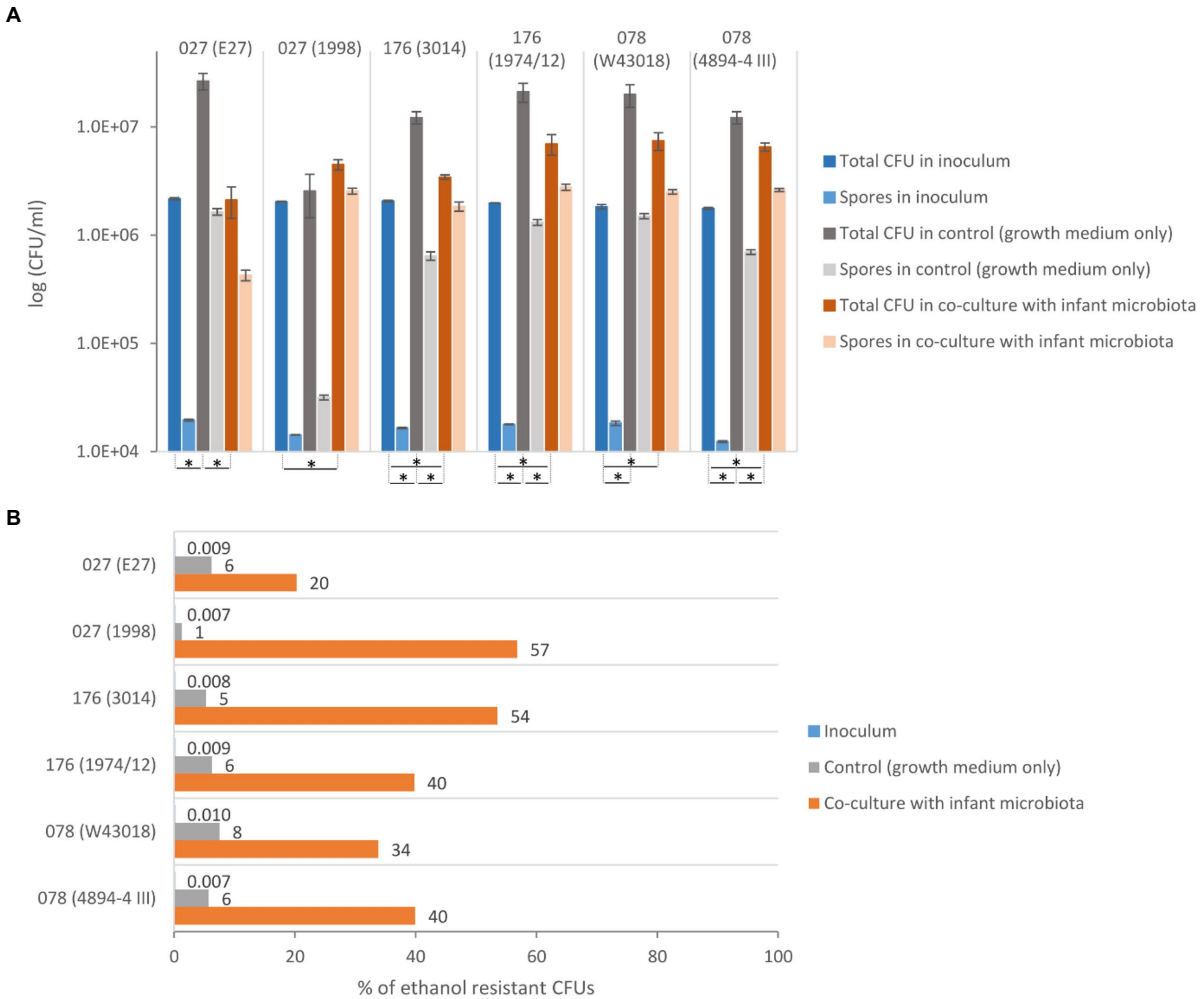
Growth and sporulation of *C. difficile* in co-culture with children fecal microbiota. **(A)** The total CFU (vegetative cells and spores) and ethanol resistant CFU (spores) with the corresponding standard deviation for *C. difficile* ribotypes 027, 176 and 078 strains. *Statistically significant difference (one-way ANOVA: *p* < 0.05). **(B)** The percentage of *C. difficile* spores detected as ethanol resistant CFUs in proportion to the total CFU for ribotypes 027, 176 and 078 strains.

Compared to the initial inoculum, a significant increase in total CFU was observed in co-culture with the children microbiota for all strains, except for the historical ribotype 027 strain E27 (Figure 1A). This indicates that *C. difficile* grows in the presence of children microbiota. Still, four out of six tested strains grew significantly better in control samples (growth medium only) than in co-cultures with children microbiota. Both strains of ribotype 078 and 176 displayed a similar pattern of growth in the absence or presence of children microbiota, whereas the growth pattern of ribotype 027 strains was different (Figure 1A).

All six strains formed a high percentage of spores (i.e., ethanol resistant CFUs) in the co-culture with children microbiota (20–57%), while in the control medium the spore percentage was lower than 8% (Figure 1B). The percentage of *C. difficile* spores in the inoculum was lower than 0.01%. The historical ribotype 027 strain had the lowest frequency of sporulation in combination with children microbiota.

### The children fecal microbiota composition and diversity are affected by the presence of *Clostridioides difficile* vegetative cells and *Clostridioides difficile* conditioned medium

A total of 2,561,939 sequences of bacterial origin were obtained with an average depth of 64,048 sequences per sample. Sequences were classified into 311 OTUs with a relative abundance higher than 0.001%. Additionally, we denoised reads into ASVs but the patterns in alpha and beta diversity between OTU and ASV analysis did not differ significantly (Supplementary Figures 1, 4). Thus, only OTU based analysis is presented in the main text.

To estimate the changes in the microbiota composition due to *in vitro* cultivation we compared the composition of the original fecal input sample community to the *in vitro* communities in control samples with the microbiota only (Supplementary Figure 2). The shift in represented phyla was evident, particularly in the loss of *Verrucomicrobia* and unclassified taxa in our *in vitro* model. On the other hand, we observed a significant increase in phylum *Bacteroidetes* (one-way ANOVA: *p* < 0.05).

The profiles of children microbiota grown in combination with *C. difficile* ribotypes 027 and 176 strains were very similar and differed from control samples, with the lower percentage of *Lachnospiraceae* and *Ruminococcaceae* (Supplementary Figures 2C,D). Microbiota co-cultured with *C. difficile* ribotype 078 displayed profiles similar to that in control samples (Supplementary Figures 2C,D). In the conditioned medium of ribotype 078 strain (designation 4,894-4III, originating from cattle) a specific microbiota pattern was noticed with a visible increase of *Clostridiaceae* compared to the control samples (Supplementary Figure 2D).

**Figure 2:**
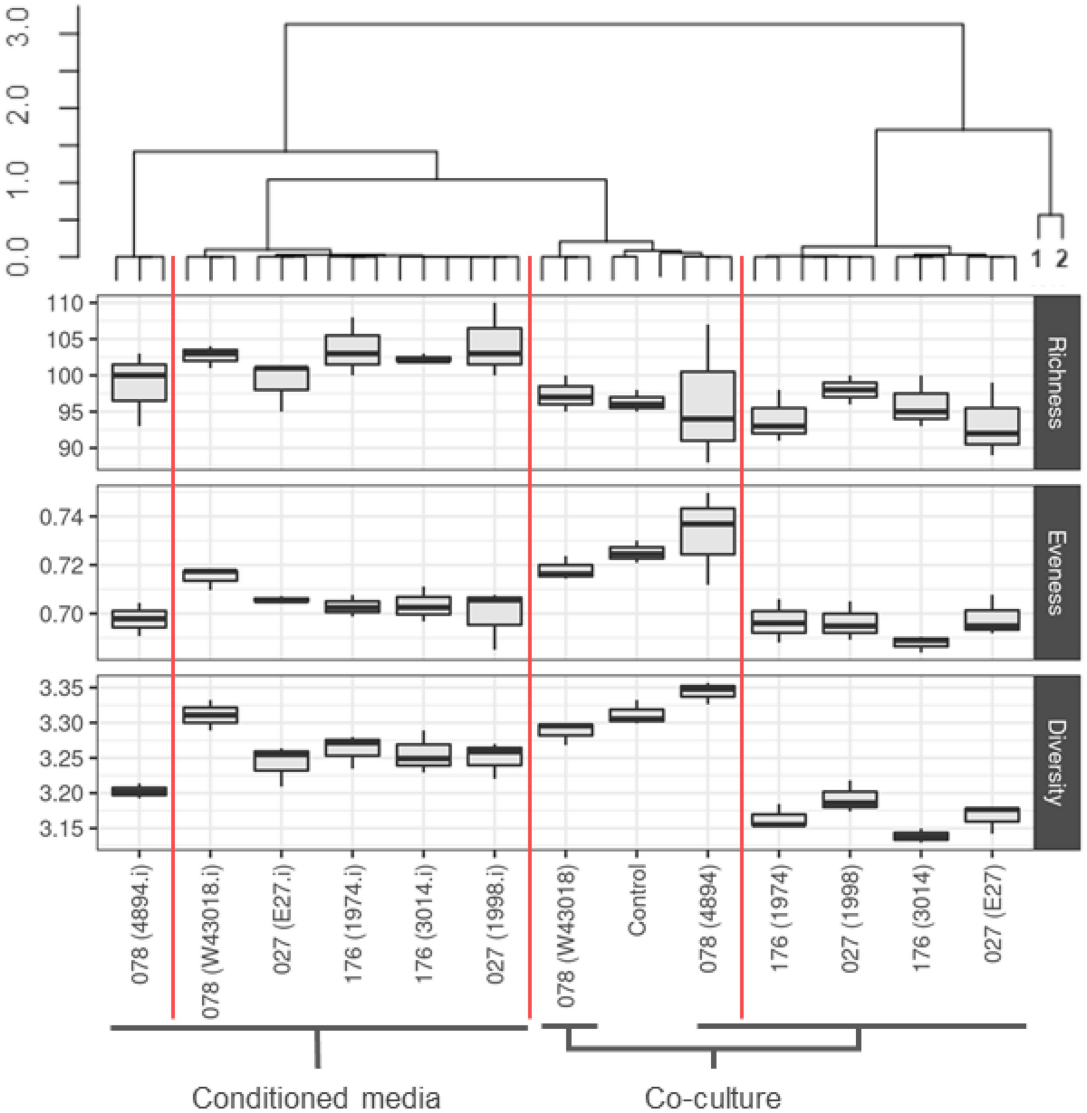
Community composition and alpha diversity of children microbiota after exposure to *C. difficile* culture or conditioned media. Clustering was performed on community compositions using correlation distances and ward. D clustering (R, pvclust package). Red vertical lines separate clusters based on 95% CI. Alpha indexes are presented with boxplots for each *C. difficile* strain, separately for co-cultures and conditioned media. We used number of unique OTUs for richness estimation, Shannon eveness for eveness estimation and Shannon for community diversity. Sampes 1 and 2 represent original fecal sample and over-night fecal culture, respectively.

Microbiota in co-cultures with *C. difficile* and microbiota grown in *C. difficile* conditioned media clustered separately (Figure 2). Most distinct characteristics were observed in microbiota co-cultured with *C. difficile* ribotypes 027 and 176. On the other hand, co-culture with ribotype 078 most closely resembled control treatment, as evident by alpha diversity measurements (Figure 2). Richness was increased in microbiota from conditioned media, while evenness was decreased under both conditions, but more in co-cultures compared to conditioned media (Figure 2). The lowest microbial diversity was observed in microbiota modulated with *C. difficile* ribotypes 027 and 176 (Figure 2).

### *Clostridioides difficile* vegetative cells and *Clostridioides difficile* conditioned media have a different impact on certain OTUs

According to AMOVA of weighted UniFrac distances, statistically significant differences in OTU composition were observed between control samples and co-cultures with *C. difficile* (*p* < 0.001) as well as between control samples and samples of children microbiota grown in *C. difficile* conditioned media (p < 0.001). We have used the LEfSe test to identify significantly changed OTUs (Supplementary Tables 1, 2). Altogether, 66 OTUs were affected in co-cultures with *C. difficile* and 77 OTUs in *C. difficile* conditioned media. Majority of significantly changed OTUs (75%) overlapped between fecal populations exposed to conditioned media and those exposed to *C. difficile* co-cultures (Supplementary Tables 1, 2).

Several OTUs reacted similarly in the presence of either *C. difficile* vegetative cells or conditioned media. For example, *Veillonella* was always decreased, while *Clostridium_XIVa* and one *Bacteroides* group were increased in the presence of *C. difficile* vegetative cells and conditioned media (Supplementary Tables 3, 4). However, some OTUs were affected differently by *C. difficile* vegetative cells and conditioned media. For instance, when compared to control samples, *Morganella* was increased in co-cultures microbiota with *C. difficile*, while it was decreased in conditioned media (Supplementary Tables 3, 4). The opposite effect was observed for *Flavonifractor*, which was increased in conditioned media and decreased in microbiota with *C. difficile* (Supplementary Tables 3, 4).

### According to ^1^H NMR spectra, samples congregate in three main clusters, which significantly differ in diversity and OTU composition

**Figure 3:**
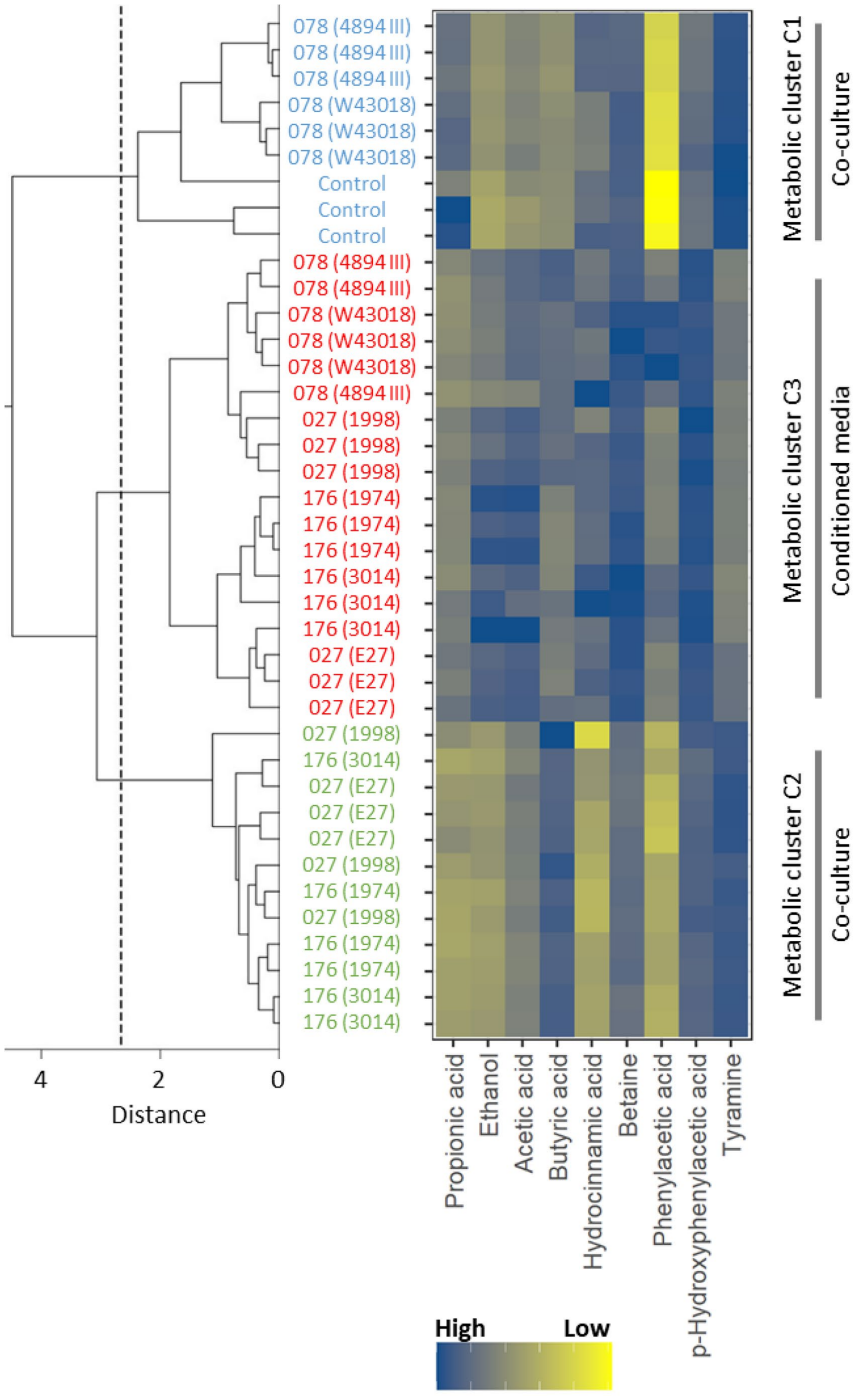
A dendrogram of the hierarchical cluster tree of NMR spectra of supernatants with respect to concentration differences of nine metabolites identified by the general explanation method. The blue color indicates the control supernatant samples of children fecal microbiota only and samples of co-cultures children fecal microbiota/*C. difficile* ribotype 078 strains (W43018, 4894; cluster 1), the green color marks the supernatant samples of co-cultures children fecal microbiota/*C. difficile* ribotypes 027 and 176 strains (E27, 1998, 3014, 1974; cluster 2) and the red color represents the supernatant samples of children fecal microbiota in *C. difficile* conditioned media (all ribotypes; cluster 3). The dashed line indicates the distance, at which the supernatant samples divide into three clusters.

The bucketed and normalized dataset of ^1^H NMR spectra of supernatants was initially analyzed with a hierarchical clustering method, which showed that supernatants congregated in three major clusters, i.e., (i) cluster 1 grouped control samples of children microbiota and samples of co-cultures microbiota with *C. difficile* ribotype 078, (ii) cluster 2 grouped samples of co-cultures microbiota with *C. difficile* ribotypes 027 and 176, and (iii) cluster 3 grouped samples of microbiota cultured in *C. difficile* conditioned media (Figure 3). The clusters of ^1^H NMR spectra of supernatants (Figure 3) correlated well with clusters based on microbiota composition (Figure 2).

To acquire information about metabolites that discriminated the three clusters, the dataset was further analyzed with the random forest model, which achieved a prediction accuracy of 100% as tested with the “leave one out” cross validation method. The perfect prediction accuracy demonstrated the ability of the random forests model to successfully learn of ^1^H NMR binned regions, which discriminated the three clusters of supernatants. Next, the general explanation method was applied to accurately highlight the most important binned regions used for predictions by the random forests model (Pecnik et al., 2018). NMR signals in the most important binned regions of ^1^H NMR spectra were assigned and used to identify nine metabolites with the use of 2D ^13^C HSQC, ^13^C HMBC and TOCSY spectra.

The normalized integral values in the binned regions of the identified metabolites that contributed significantly to the clustering of supernatants are shown in Supplementary Figure 3. Since integral values are directly proportional to the metabolite concentration, it is apparent that higher concentrations of tyramine were found in clusters 1 and 2, whereas lower levels were associated with samples of cluster 3 (Figure 3). Phenylacetic acid and p-hydroxyphenylacetic acid were present in lower to medium concentrations in clusters 1 and 2, while in samples of cluster 3 higher levels were observed. The opposite was detected for acetic acid. In samples with higher concentrations of acetic acid (i.e., cluster 1 and 2) lower levels of ethanol were detected. Higher concentrations of hydrocinnamic acid were observed for clusters 1 and 3. All three clusters have different concentrations of propionic acid and betaine. Cluster 1 exhibits higher, cluster 2 lower and cluster 3 medium concentrations of propionic acid. On the other hand, higher concentrations of betaine can be observed for cluster 3, lower for cluster 2 and medium for cluster 1. Furthermore, the presence of *C. difficile* (vegetative cells and conditioned media) was associated with high concentration of butyric acid, one of the *C. difficile* metabolic products.

**Figure 4:**
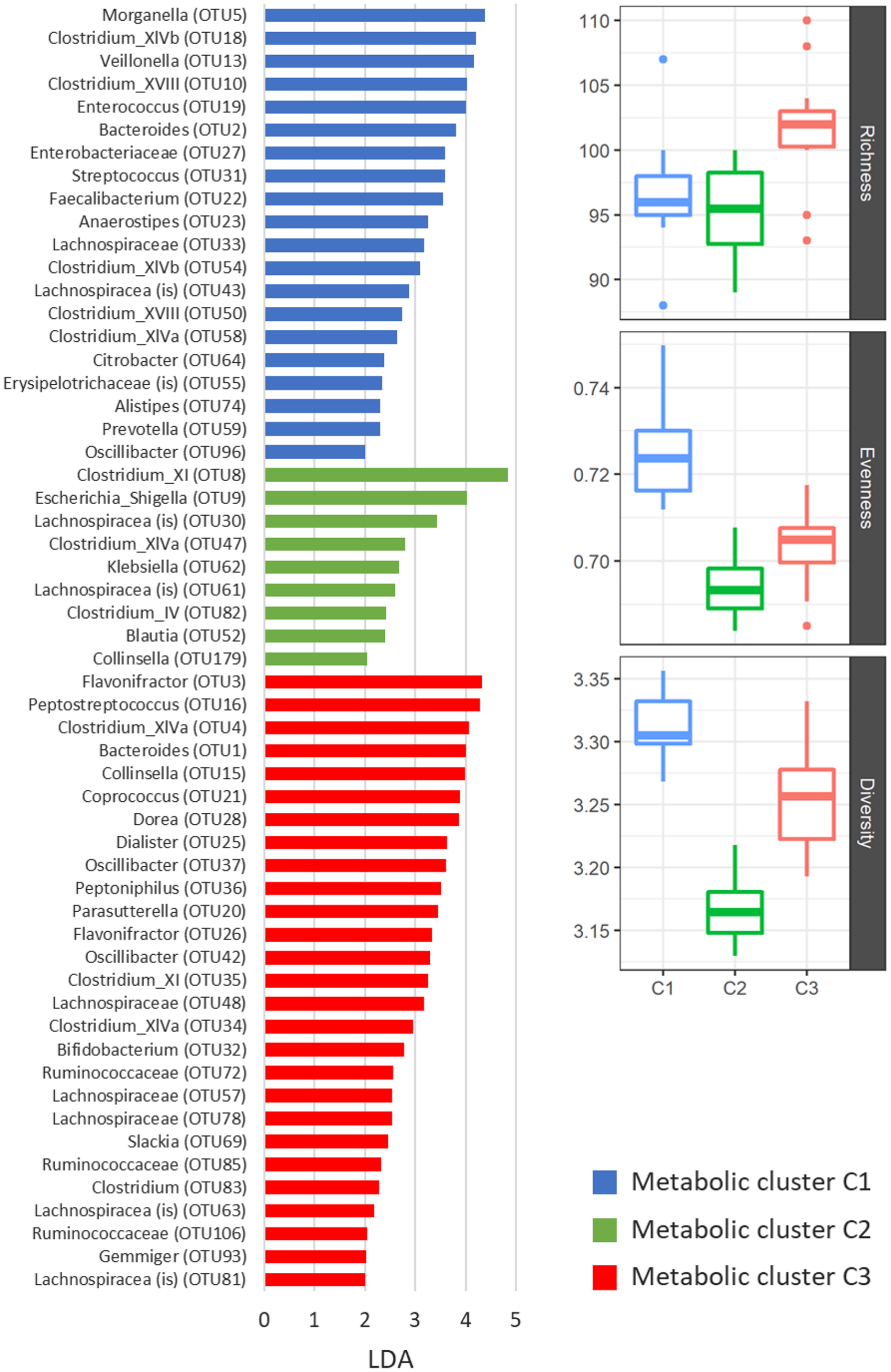
Differentially represented OTUs in three clusters of NMR spectra of supernatants. The presented OTUs were identified by the LEfSe test (mothur software), which uses linear discriminant analysis (LDA) to find OTUs, which significantly differ in abundance between all groups of samples. Alpha indexes are presented with boxplots for each metabolic cluster separately. We used number of unique OTUs for richness estimation, Shannon eveness for eveness estimation and Shannon for community diversity.

Microbiota composition differed significantly between metabolic clusters (Figure 4). According to the AMOVA analysis on microbial communities, statistically significant differences in OTU composition were observed among all three clusters (*p* < 0.001). Comparable to microbiota diversity-associated clustering (Figure 2) we also observed similarities in metabolic profiles between control samples and co-culture with ribotype 078 (Figure 4). This cluster had a significantly higher bacterial diversity (one-way ANOVA: *p* < 0.05; Figure 4) and several overrepresented taxa (Figure 4, blue columns). The lowest bacterial diversity was noticed in cluster 2, containing samples of children microbiota co-cultures with *C. difficile* ribotypes 027 and 176. Here, an increased abundances of *Clostridium* XI (including *C. difficile*), *Escherichia_Shigella* and *Klebsiella* were observed.

## Discussion

In the current study we aimed to test interactions between *C. difficile* and the children’s gut microbiota. Compared to a generally stable and complex adult gut microbiota, which provides a colonization resistance against *C. difficile*, the children’s gut microbiota is dynamic and structurally simple. Along with maturation of gut microbiota, *C. difficile* colonization in children decreases to 10% at the age of 2 years (Rousseau et al., 2011; Davis et al., 2016; Smith et al., 2020).

In our previous study (Horvat and Rupnik, 2018) the interactions between *C. difficile* and healthy adult or disrupted adult microbiota were investigated. The results of the previous and current study cannot be directly compared, as used *C. difficile* strains were different, except of ZZV09-2033. Despite this, we observed similar patterns in both studies. Namely, *C. difficile* sporulation was very high in co-culture with disrupted/undeveloped gut microbiota, either from adults (Horvat and Rupnik, 2018) or children (current study). High sporulation rate in the presence of children gut microbiota *in vitro* is suggestive of high sporulation also *in vivo*. This would imply that colonized children could shed the spores very effectively and could explain why a contact with children below 2 years of age is reported as a risk factor for CDI (Stoesser et al., 2017; Crobach et al., 2018).

There were no major differences in growth/sporulation patterns for strains belonging to the same ribotype, except for ribotype 027 strains. One of the two included 027 strains is the historical non-epidemic strain (here designated as ZZV09-2033 and generally known as CD 196; Perelle et al., 1997). This strain showed the poorest growth and sporulation in co-culture with children microbiota. Our results suggest that the epidemic 027 strain could overcome colonization resistance more successfully than non-epidemic historic 027 strain (Figure 1). These findings are in line with observations that epidemic 027 strains utilize several nutritional sources (Scaria et al., 2014) and/or successfully compete for the sources even in the presence of a complex microbiota (Robinson et al., 2014). This could also partially explain their widespread isolation on a global scale (Martin et al., 2016).

Effects of *C. difficile* on fecal microbiota were tested either in the presence of vegetative *C. difficile* cells (co-culture *C. difficile*/microbiota) or in cultivation in cell free spent *C. difficile* medium. Microbe-microbe interactions can involve direct (cell contact dependent) and indirect (metabolic inhibitory products or resource competition) mechanisms. Our two approaches address both. We also suggest that these two approaches mimics two ecological settings during *C. difficile* colonization/infection. Co-culture is similar to interaction in which *C. difficile* is not the major component in the gut, resembling the colonization stage. Fecal microbiota co-cultivation in spent medium potentially mirror stages during CDI where *C. difficile* flourish in the gut and strongly affect the metabolic gut environment.

In the co-culture with *C. difficile* and in the conditioned medium the microbial diversity of children fecal microbiota decreased and this was most obvious with ribotypes 027 and 176 (Figure 2). The changes in diversity included a decrease in *Firmicutes*, particularly the butyrate-producing members of *Ruminococcaceae* and *Lachnospiraceae* and increased abundance of *Proteobacteria* members (*Escherichia, Klebsiella*) in microbiota exposed to *C. difficile.* Similar changes were described in several other studies in adult and infant CDI (Vincent and Manges, 2015; Vasilescu et al., 2022) and apparent in our previous studies on interactions between *C. difficile* with adult disrupted and/or healthy microbiota (Horvat et al., 2017; Horvat and Rupnik, 2018). However, at least one study of children microbiome associated with *C. difficile* colonization reported contrary results and *Lachnospiraceae and Ruminococcaceae* were enriched in breastfed or formula fed *C. difficile* positive children (Drall et al., 2019).

As reported recently, differences in the microbial composition during CDI are accompanied by alterations in fecal metabolites (Rojo et al., 2015; Allegretti et al., 2016; Antharam et al., 2016; Martinez-Gili et al., 2020; Gao et al., 2022; Wan et al., 2022). Important metabolites, that differentiate among healthy controls and patients with CDI, are bile acids (Allegretti et al., 2016), sterols (Antharam et al., 2016), amino acids and some vitamins (Rojo et al., 2015; Hryckowian et al., 2017). According to our ^1^H NMR spectra analysis, critical metabolic pathways in the children microbiota affected by *C. difficile* included microbial fermentation of carbohydrates into ethanol or short chain fatty acids (SCFAs; i.e. acetic acid, butyric acid and propionic acid), fermentation of aromatic amino acids tyrosine and phenylalanine into phenolic acids (i.e., phenylacetic acid, p-hydroxyphenylacetic acid and hydrocinnamic acid) and decarboxylation of tyrosine into tyramine. High to medium concentrations of acetic acid, butyric acid, phenylacetic acid, p-hydroxyphenylacetic acid and tyramine were detected in supernatants of microbiota co-cultured with *C. difficile* vegetative cells of ribotypes 027 and 176 strains (Figure 3). Under the same experimental conditions, the lowest microbial diversity and the presence of opportunistic enterobacteria (i.e., *Escherichia, Klebsiella*) was observed (Figure 4). High concentrations of butyric acid, ethanol and phenolic acids were observed after fecal microbiota cultivation in conditioned medium (Figure 3). This cultivation was associated with the greatest perturbation of microbiota composition, according to the highest proportion of OTUs affected (Figure 4; Supplementary Figure 4; Supplementary Table 2). Results also partly reflect the *C. difficile* metabolism, which typically produces acetic and butyric acids, whereas propionic acid and phenolic acids are usually produced in trace amounts (Engelkirk et al., 1992).

Although beneficial to colonic health, the accumulation of SCFAs can cause a drop in the colonic pH, which inhibits bacterial transformation of primary bile acids (i.e., taurocholate) into secondary bile acids (i.e., deoxycholate; Wong et al., 2006). These changes are observed after antibiotic treatment and promote *C. difficile* outgrowth (Schnizlein and Young, 2022). In addition, butyric acid can induce *C. difficile* toxin production (Karlsson et al., 2000), which causes inflammation that could further support *C. difficile* growth in the gut (Hryckowian et al., 2017). In contrast, *in vitro* models showed that acetate and propionate can inhibit *C. difficile* toxin production (Gao et al., 2022). Ethanol is another product of carbohydrate fermentation, which, if present in high concentrations, can cause intestinal dysbiosis (Engen et al., 2015), whereas phenolic acids have proven antimicrobial properties against several commensal gut bacteria (Cueva et al., 2010). 3-Phenillacetic acid was among significantly increased metabolites in patients with Ulcerative colitis and CDI (Wan et al., 2022). Tyramine is a bio-active catabolite of amino acid tyrosine, which can be used as a carbon and nitrogen source for gut enterobacteria, particularly *Escherichia* (Diaz et al., 2001), and could therefore contribute to their expansion during CDI.

We also acknowledge that our study has a few limitations. Use of pooled fecal samples rather than individual samples might have resulted in more variable results due to diverse *C. difficile* strains. However, we aimed to investigate effects of different *C. difficile* strains, therefore the use of pooled samples was most appropriate. This largely limits the comparisons of the effects between different microbiota types (adult and children) and *C. difficile*. Finally, we are using a very simple batch system, while a chemostat system would enable longer time periods for analysis. However, the batch system enables analysis of more strain/microbiota type combinations. Although many mechanisms of colonization resistance towards *C. difficile* are elucidated, there are still open questions about mutual interplay between *C. difficile* and the gut ecosystem. The underlying mechanisms of observed changes are one of the main aims of our future studies. Further investigation on potential differences between strains of human and animal origin would be also warranted, as here animal strains of ribotype 078 affected microbiota differently to human ribotype 78 strains.

In summary, our results showed that *C. difficile* caused changes in children gut microbiota and metabolome in an *in vitro* model. Microbiota metabolic clusters were differentiated after growth with *C. difficile* or exposure to *C. difficile* conditioned medium. The differentiating metabolites (SCFA, ethanol, phenolic acids, tyramine) have known effects on protective gut microbiota members and *C. difficile* biology, potentially contributing to *C. difficile* colonization. In addition, the increased *C. difficile* sporulation rates in the presence of children’s microbiota was observed. This finding could suggest high spore loads in children also *in vivo* which is in agreement with observations that contact with children is a risk factor for CDI.

## Data availability statement

The sequence data was deposited on the Metagenomics RAST (MG-RAST) database server1 under the project access number mgp90153.

## Ethics statement

The studies involving human participants were reviewed and approved by Ethical Committee, Medical Faculty, University of Maribor. Written informed consent to participate in this study was provided by the participants’ legal guardian/next of kin.

## Author contributions

SH and MR designed the study. SH performed microbiological experiments. DM, KP, and JP performed NMR analysis. SH, AM, DM, and KP performed the data analysis. MR and JP contributed to the data analysis. All authors contributed to the article and approved the submitted version.

## Funding

The authors acknowledge the financial support from the Slovenian Research Agency (young researchers program, P3-0387 and P1-0242).

## Conflict of interest

The authors declare that the research was conducted in the absence of any commercial or financial relationships that could be construed as a potential conflict of interest.

## Publisher’s note

All claims expressed in this article are solely those of the authors and do not necessarily represent those of their affiliated organizations, or those of the publisher, the editors and the reviewers. Any product that may be evaluated in this article, or claim that may be made by its manufacturer, is not guaranteed or endorsed by the publisher.

## Footnotes

^1^ http://metagenomics.anl.gov/

^2^ https://www.mg-rast.org/linkin.cgi?project=mgp90153
