# Supplementary material for: Children gut microbiota exhibits a different composition and metabolic profile after in vitro exposure to Clostridioides difficile and increases its sporulation
Source: Front Microbiol. 2022 Dec 9;13:1042526. doi: 10.3389/fmicb.2022.1042526 (PMC9780542; doi:10.3389/fmicb.2022.1042526)
Supplement: Supplementary file 1 [file Data_Sheet_1.PDF]

Supplementary Material

Children gut microbiota exhibits a different composition and metabolic profile after exposure to *Clostridioides difficile* and increases its sporulation

Sabina Horvat, Aleksander Mahnic, Damjan Makuc, Klemen Pečnik, Janez Plavec, Maja Rupnik*

***Correspondence**: Corresponding Author: maja.rupnik@nlzoh.si

OTUs (mothur)

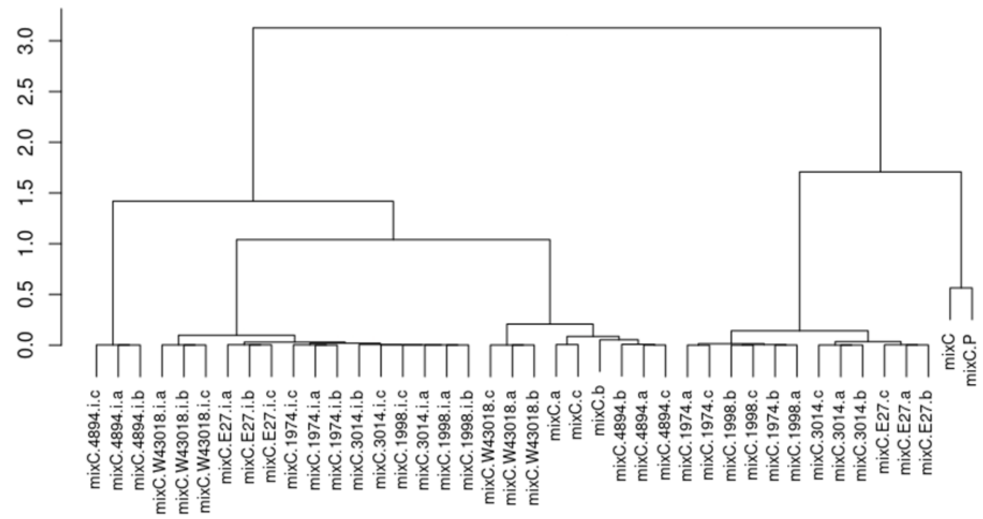

ASVs (Usearch)

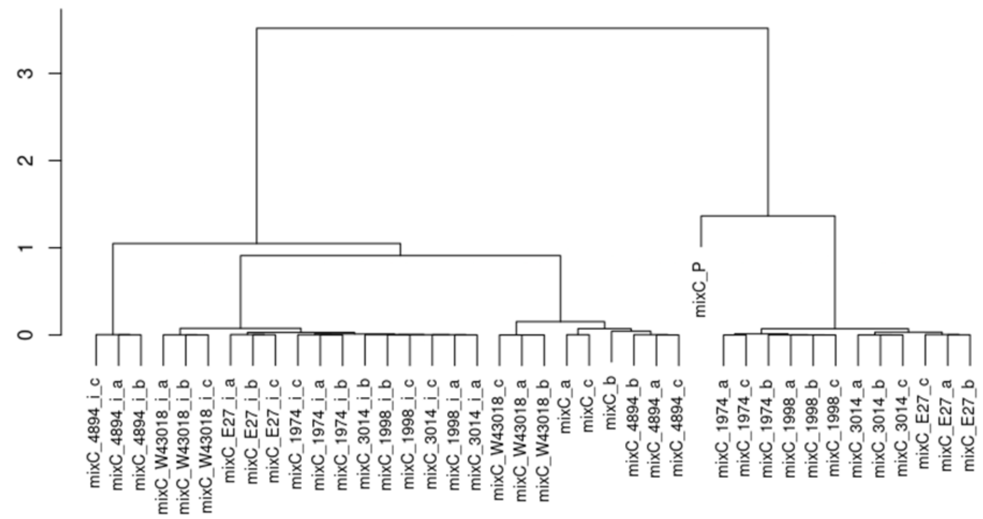

Supplementary Figure 1: Hierarchical clustering of samples, comparison between OTUs and ZOTUs. Clustering was performed separately on OTUs (mothur, v.1.36.1) and ASVs (Usearch, v11.0.667) using correlation distance method and ward.D clustering (R, pvclust package). Clustering shows large overlap of both read binning methods.

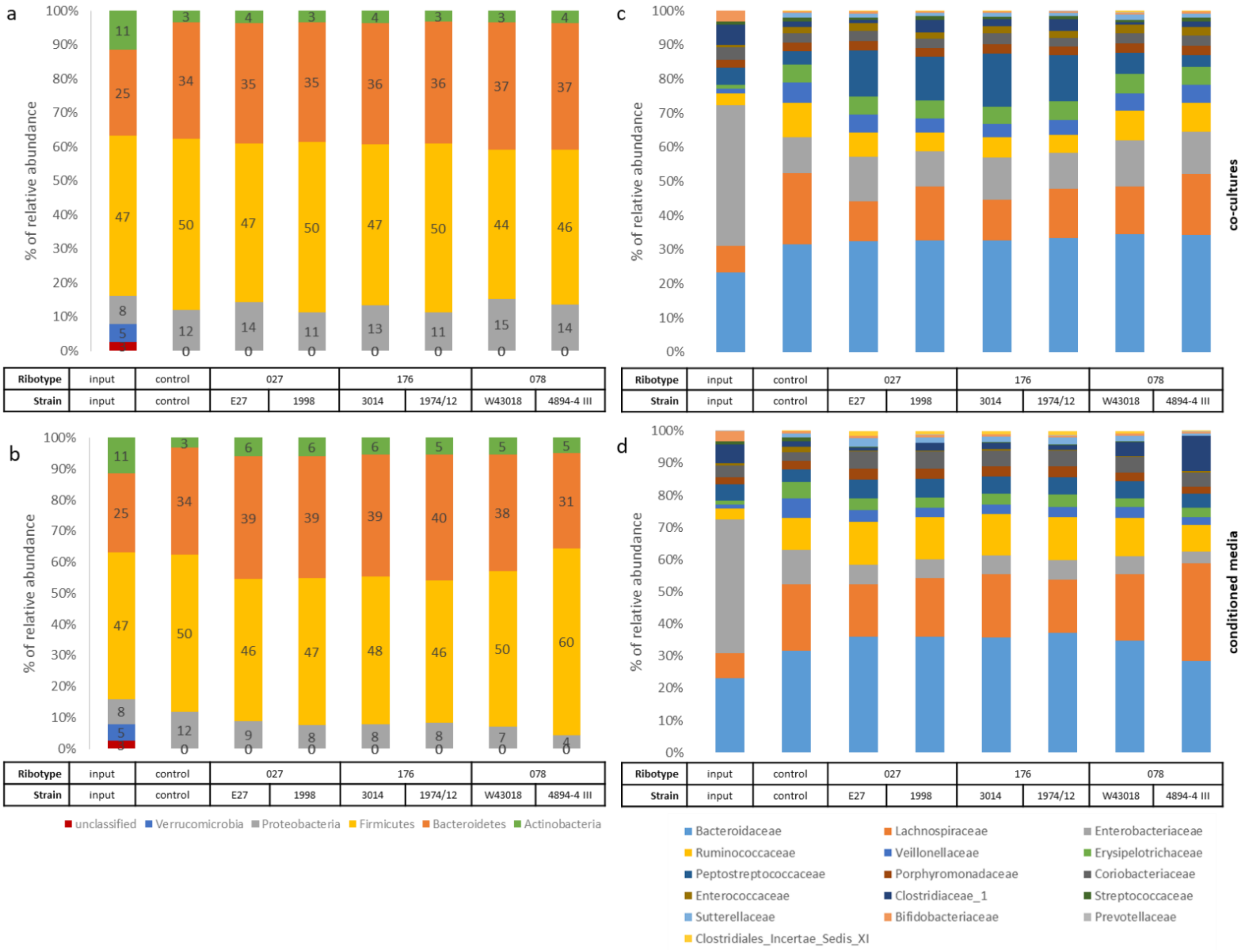

Supplementary Figure 2: Bacterial phylum (a and b) and family (c and d) level assignments of average relative abundances for children fecal input samples (before any cultivation), control samples (children fecal microbiota only) and samples of co-cultures childern fecal microbiota/*C. difficile* ribotypes 027, 176 and 078 strains (a and c) or samples of children fecal microbiota cultured in conditioned media of *C. difficile* ribotypes 027, 176 and 078 strains (b and d).

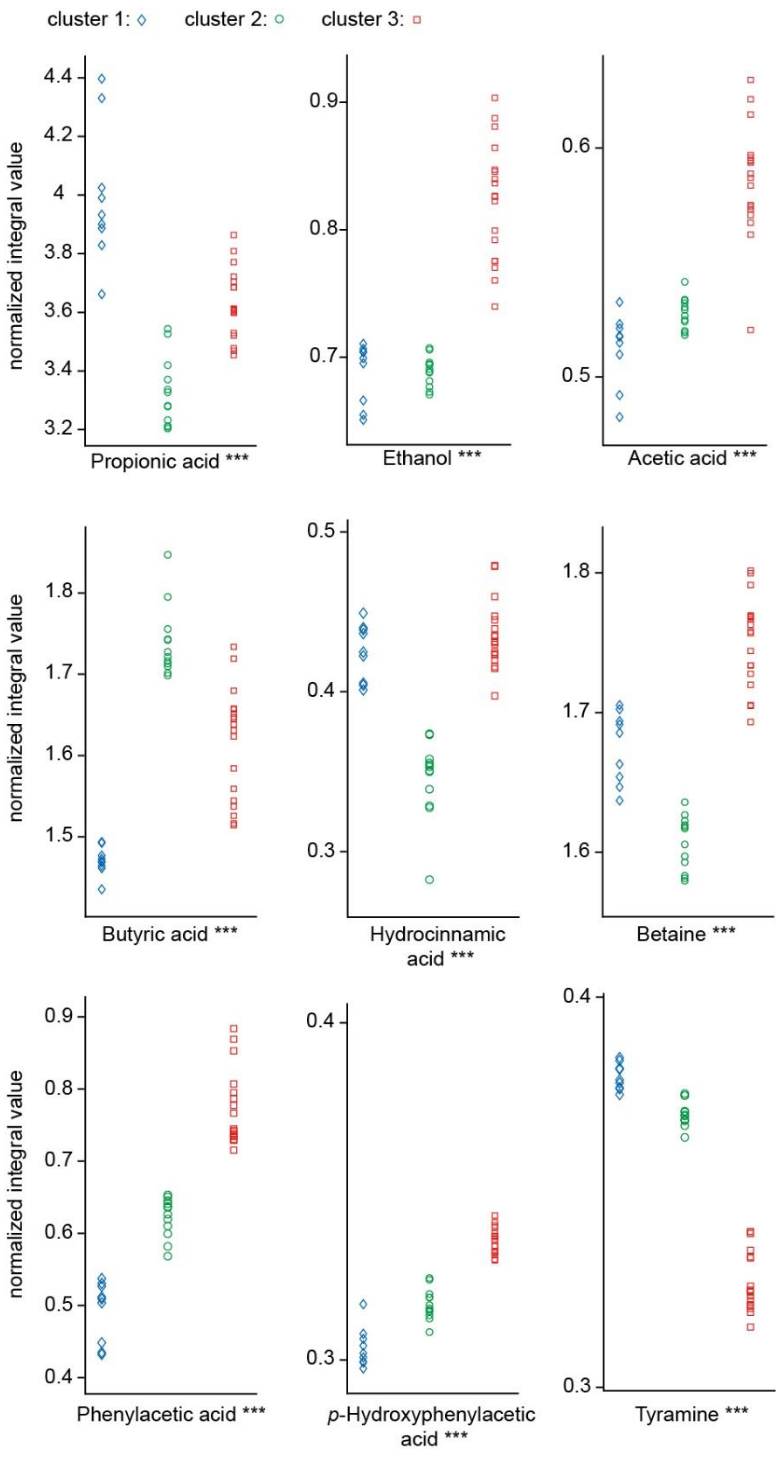

Supplementary Figure 3: Normalized integral values of 9 important binned regions for the ANN model for all samples in the three clusters. Cluster classes are indicated at the top. ***p<0.001, one-way ANOVA.

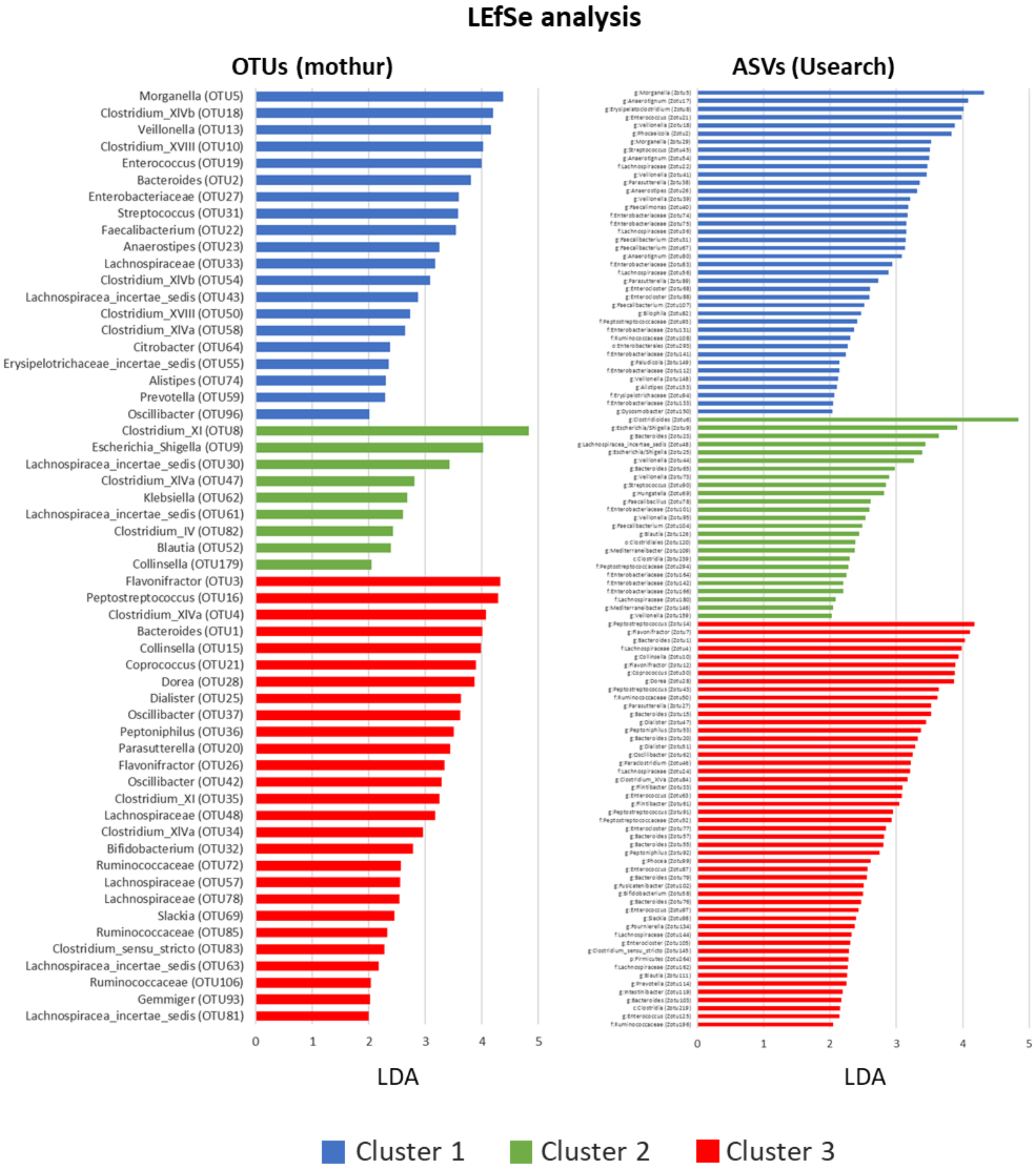

Supplementary Figure 4: Differentially represented taxa between metabolome-based clusters, comparison between OTUs and ASVs. LEfSe analysis was performed on both OTUs (mothur, v.1.36.1) and ASVs (Usearch, v11.0.667). Only taxa that were significant after Benjamini-Hochberg correction (FDR < 0.05) are presented. We obtained 115 (42.8% of total) differentially represented ASVs compared to 56 OTUs (29.5% of total). However, most significant differentially represented taxa coincide on the genus taxonomic level between both methods.

Supplementary Table 1: OTUs that significantly differ in abundance between cultures of children fecal microbiota only (control) and co-cultures of children fecal microbiota with *C. difficile* ribotypes 027 (strains E27, 1998), 176 (strains 3014 and 1974) and 078 (strains W43018 and 4894), identified by the LEfSe test (mothur software).

comparison	control-E27	control-1998	control-3014	control-1974	control-W43018	control-4894			
WUniFrac	< 0.001	< 0.001	< 0.001	< 0.001	< 0.001	< 0.001	Phylum	Family	Genus
Otu00003	4.08	4.30	4.24	4.32	3.82	3.80	*Firmicutes*	*Ruminococcaceae*	*Flavonifractor*
Otu00016	4.12	4.13	4.15	4.18	3.88	3.88	*Firmicutes*	*Peptostreptococcaceae*	*Peptostreptococcus*
Otu00022	3.75	3.69	3.75	3.71	3.67	3.41	*Firmicutes*	*Lachnospiraceae*	*Coprococcus*
Otu00029	3.46	3.19	3.41	3.28	3.50	3.05	*Firmicutes*	*Streptococcaceae*	*Streptococcus*
Otu00013	0.00	3.84	3.94	3.76	3.66	3.81	*Firmicutes*	*Veillonellaceae*	*Veillonella*
Otu00043	3.32	3.22	3.36	3.34	0.00	3.10	*Firmicutes*	*Ruminococcaceae*	*Oscillibacter*
Otu00031	3.07	3.26	3.30	3.35	2.75	0.00	*Firmicutes*	*Lachnospiraceae*	*Dorea*
Otu00059	3.14	3.11	3.20	3.14	3.06	0.00	*Firmicutes*	*Lachnospiraceae*	*Clostridium_XlVb*
Otu00154	2.46	2.41	2.76	2.48	2.45	2.30	*Firmicutes*	*Ruminococcaceae*	uncl. from *Ruminococcaceae*
Otu00045	2.93	2.72	2.89	2.92	2.81	0.00	*Firmicutes*	*Lachnospiraceae*	*Lachnospiracea_incertae_sedis*
Otu00140	2.31	2.29	2.40	2.40	2.29	2.28	*Firmicutes*	*Ruminococcaceae*	uncl. from *Ruminococcaceae*
Otu00130	2.25	2.17	2.49	2.41	2.06	2.17	*Firmicutes*	*Ruminococcaceae*	*Oscillibacter*
Otu00068	2.64	2.71	2.91	2.79	0.00	2.46	*Firmicutes*	*Lachnospiraceae*	*Clostridium_XlVa*
Otu00012	4.41	0.00	4.31	0.00	4.43	0.00	*Firmicutes*	*Lachnospiraceae*	uncl. from *Lachnospiraceae*
Otu00065	2.22	2.18	2.51	2.42	2.24	0.00	*Bacteroidetes*	*Rikenellaceae*	*Alistipes*
Otu00064	2.26	2.32	0.00	2.47	2.16	2.31	*Firmicutes*	*Lachnospiraceae*	uncl. from *Lachnospiraceae*
Otu00025	3.45	3.43	3.41	3.46	0.00	-3.09	*Firmicutes*	*Veillonellaceae*	*Dialister*
Otu00038	2.51	2.68	2.74	2.70	-2.46	0.00	*Firmicutes*	*Ruminococcaceae*	*Oscillibacter*
Otu00002	0.00	3.59	3.59	3.57	-3.32	0.00	*Bacteroidetes*	*Bacteroidaceae*	*Bacteroides*
Otu00164	0.00	0.00	2.59	2.29	0.00	2.06	*Firmicutes*	*Ruminococcaceae*	uncl. from *Ruminococcaceae*
Otu00091	0.00	2.09	2.66	0.00	0.00	2.08	*Firmicutes*	*Lachnospiraceae*	uncl. from *Lachnospiraceae*
Otu00020	0.00	3.24	3.16	3.30	-3.34	0.00	*Proteobacteria*	*Sutterellaceae*	*Parasutterella*
Otu00030	0.00	3.12	2.80	3.18	-2.89	0.00	*Proteobacteria*	*Enterobacteriaceae*	uncl. from *Enterobacteriaceae*
Otu00071	0.00	2.45	2.65	2.50	-2.31	0.00	*Proteobacteria*	*Enterobacteriaceae*	*Citrobacter*
Otu00035	0.00	2.66	0.00	2.55	0.00	0.00	*Firmicutes*	*Lachnospiraceae*	*Blautia*
Otu00019	0.00	4.38	0.00	0.00	0.00	0.00	*Firmicutes*	*Lachnospiraceae*	*Clostridium_XlVb*
Otu00041	2.71	-2.70	2.80	-2.33	2.73	0.00	*Firmicutes*	*Clostridiaceae*	*Clostridium_sensu_stricto*
Otu00014	0.00	0.00	0.00	3.04	0.00	0.00	*Actinobacteria*	*Coriobacteriaceae*	*Collinsella*
Otu00032	0.00	0.00	0.00	2.81	0.00	0.00	*Firmicutes*	*Lachnospiraceae*	*Clostridium_XlVa*
Otu00151	0.00	0.00	2.65	0.00	0.00	0.00	*Firmicutes*	*Lachnospiraceae*	uncl. from *Lachnospiraceae*
Otu00080	0.00	0.00	0.00	0.00	2.22	0.00	*Actinobacteria*	*Coriobacteriaceae*	*Slackia*
Otu00076	2.22	0.00	0.00	0.00	0.00	0.00	*Bacteroidetes*	*Prevotellaceae*	*Prevotella*
Otu00070	2.21	0.00	0.00	0.00	0.00	0.00	*Proteobacteria*	*Enterobacteriaceae*	*Klebsiella*
Otu00149	0.00	0.00	0.00	2.20	0.00	0.00	*Firmicutes*	*Ruminococcaceae*	uncl. from *Ruminococcaceae*
Otu00063	0.00	0.00	0.00	2.16	0.00	0.00	*Firmicutes*	*Erysipelotrichaceae*	*Erysipelotrichaceae_incertae_sedis*
Otu00105	0.00	0.00	0.00	2.12	0.00	0.00	*Firmicutes*	*Clostridiales_Incertae_Sedis_XI*	*Peptoniphilus*
Otu00134	0.00	0.00	0.00	2.03	0.00	0.00	*Firmicutes*	*Ruminococcaceae*	*Oscillibacter*
Otu00067	2.07	-2.18	0.00	-2.39	0.00	0.00	*Firmicutes*	*Ruminococcaceae*	*Butyricicoccus*
Otu00048	0.00	2.11	0.00	0.00	-2.36	-2.55	*Firmicutes*	*Lachnospiraceae*	*Roseburia*
Otu00018	0.00	0.00	0.00	0.00	0.00	-3.18	*Firmicutes*	*Ruminococcaceae*	*Faecalibacterium*
Otu00077	0.00	0.00	-2.53	0.00	0.00	-2.08	*Firmicutes*	*Lachnospiraceae*	*Lachnospiracea_incertae_sedis*
Otu00015	3.41	-4.04	-3.54	-3.99	3.50	0.00	*Firmicutes*	*Clostridiaceae*	*Clostridium_sensu_stricto*
Otu00087	-2.55	0.00	-2.88	0.00	0.00	0.00	*Firmicutes*	*Ruminococcaceae*	uncl. from *Ruminococcaceae*
Otu00033	0.00	2.87	-2.59	0.00	-3.11	-2.97	*Firmicutes*	*Peptostreptococcaceae*	*Clostridium_XI*
Otu00136	0.00	-2.07	0.00	-2.07	0.00	-2.12	*Firmicutes*	*Clostridiales_Incertae_Sedis_XI*	*Finegoldia*
Otu00011	3.37	-3.15	0.00	-3.51	0.00	-3.39	*Bacteroidetes*	*Bacteroidaceae*	*Bacteroides*
Otu00051	0.00	-2.37	0.00	0.00	-2.53	-2.50	*Firmicutes*	*Erysipelotrichaceae*	*Clostridium_XVIII*
Otu00109	0.00	-2.14	-3.09	-2.20	0.00	0.00	*Firmicutes*	*Lachnospiraceae*	uncl. from *Lachnospiraceae*
Otu00006	-3.59	3.56	-3.62	3.65	-4.00	-3.87	*Proteobacteria*	*Enterobacteriaceae*	*Morganella*
Otu00026	-2.67	0.00	-2.84	0.00	-2.51	0.00	*Firmicutes*	*Lachnospiraceae*	*Dorea*
Otu00056	-2.42	-2.57	-2.55	-2.73	2.21	0.00	*Firmicutes*	*Lachnospiraceae*	*Blautia*
Otu00039	-2.83	0.00	0.00	0.00	-3.31	-2.25	*Firmicutes*	*Clostridiales_Incertae_Sedis_XI*	*Peptoniphilus*
Otu00066	0.00	-2.71	-2.90	-2.86	0.00	0.00	*Firmicutes*	*Lachnospiraceae*	*Lachnospiracea_incertae_sedis*
Otu00079	0.00	-2.43	-2.74	-2.33	-2.08	-2.17	*Firmicutes*	*Ruminococcaceae*	uncl. from *Ruminococcaceae*
Otu00034	-3.15	-2.99	0.00	0.00	-3.02	-3.02	*Firmicutes*	*Lachnospiraceae*	*Lachnospiracea_incertae_sedis*
Otu00023	-2.93	0.00	-3.17	0.00	-3.23	-3.16	*Firmicutes*	*Lachnospiraceae*	*Anaerostipes*
Otu00027	-2.36	-2.96	0.00	-2.76	-2.47	-2.70	*Firmicutes*	*Ruminococcaceae*	*Flavonifractor*
Otu00021	-3.30	0.00	-3.11	0.00	-3.46	-3.43	*Firmicutes*	*Enterococcaceae*	*Enterococcus*
Otu00017	-3.05	0.00	-3.12	-2.92	-3.17	-3.06	*Bacteroidetes*	*Porphyromonadaceae*	*Parabacteroides*
Otu00004	-3.91	-3.68	-3.71	0.00	-4.11	0.00	*Firmicutes*	*Lachnospiraceae*	*Clostridium_XlVa*
Otu00024	-3.27	-2.89	-3.24	0.00	-3.22	-3.22	*Actinobacteria*	*Coriobacteriaceae*	*Eggerthella*
Otu00050	-2.71	-2.98	-2.89	-3.05	-2.51	-2.43	*Firmicutes*	*Lachnospiraceae*	*Clostridium_XlVa*
Otu00005	-3.46	-2.99	-3.08	-3.57	-3.49	0.00	*Bacteroidetes*	*Bacteroidaceae*	*Bacteroides*
Otu00007	-3.35	0.00	-3.35	-3.30	-3.49	-3.42	*Bacteroidetes*	*Bacteroidaceae*	*Bacteroides*
Otu00008	-3.94	-3.52	-3.79	-3.73	-3.59	0.00	*Proteobacteria*	*Enterobacteriaceae*	*Escherichia_Shigella*
Otu00001	0.00	-3.83	-3.77	-3.58	-3.83	-3.73	*Bacteroidetes*	*Bacteroidaceae*	*Bacteroides*

OTUs with negative LDA scores (red) are enriched in samples of children fecal microbiota with added *C. difficile*, while OTUs with positive LDA scores (blue) are enriched in samples of children fecal microbiota only. OTUs are listed according to LDA score in a descending manner.

uncl., unclassified; WUniFra, weighted UniFrac; E27 and 1998, ribotype 027 strains; 3014 and 1974, ribotype 176 strains; W43018 and 4894, ribotype 078 strains

Supplementary Table 2: OTUs that significantly differ in abundance between cultures of children fecal microbiota only (control) and cultures of children fecal microbiota in conditioned media of *C. difficile* ribotypes 027 (strains E27, 1998), 176 (strains 3014 and 1974) and 078 (strains W43018 and 4894), identified by the LEfSe test (mothur software).

comparison	control-E27i	control-1998i	control-3014i	control-1974i	control-W43018i	control-4894i			
WUniFrac	< 0.001	< 0.001	< 0.001	< 0.001	< 0.001	< 0.001	Phylum	Family	Genus
Otu00013	4.20	4.27	4.28	4.23	4.20	4.27	*Firmicutes*	*Veillonellaceae*	*Veillonella*
Otu00006	4.23	4.24	4.22	4.18	4.25	4.34	*Proteobacteria*	*Enterobacteriaceae*	*Morganella*
Otu00010	3.93	3.98	3.92	3.80	4.08	4.03	*Firmicutes*	*Erysipelotrichaceae*	*Clostridium_XVIII*
Otu00021	3.88	3.89	3.88	3.89	3.91	3.89	*Firmicutes*	*Enterococcaceae*	*Enterococcus*
Otu00029	3.72	3.73	3.73	3.73	3.75	3.74	*Firmicutes*	*Streptococcaceae*	*Streptococcus*
Otu00030	3.55	3.54	3.56	3.52	3.59	3.61	*Proteobacteria*	*Enterobacteriaceae*	uncl. from *Enterobacteriaceae*
Otu00018	3.20	3.31	3.31	3.38	3.20	3.49	*Firmicutes*	*Ruminococcaceae*	*Faecalibacterium*
Otu00034	3.26	3.26	3.16	3.13	3.12	3.22	*Firmicutes*	*Lachnospiraceae*	*Lachnospiracea_incertae_sedis*
Otu00008	0.00	3.44	3.47	3.45	3.70	3.85	*Proteobacteria*	*Enterobacteriaceae*	*Escherichia_Shigella*
Otu00012	4.47	4.47	4.45	4.48	4.31	-4.62	*Firmicutes*	*Lachnospiraceae*	uncl. from *Lachnospiraceae*
Otu00070	2.73	2.70	2.62	2.68	2.67	2.75	*Proteobacteria*	*Enterobacteriaceae*	*Klebsiella*
Otu00059	2.97	3.08	3.05	3.11	2.77	0.00	*Firmicutes*	*Lachnospiraceae*	*Clostridium_XlVb*
Otu00154	2.71	2.32	2.39	2.38	2.51	2.39	*Firmicutes*	*Ruminococcaceae*	uncl. from *Ruminococcaceae*
Otu00065	2.64	2.33	2.45	2.38	2.37	2.38	*Bacteroidetes*	*Rikenellaceae*	*Alistipes*
Otu00106	2.52	2.26	2.28	2.29	2.37	2.34	*Firmicutes*	*Ruminococcaceae*	uncl. from *Ruminococcaceae*
Otu00068	0.00	2.54	2.64	2.65	2.69	2.84	*Firmicutes*	*Lachnospiraceae*	*Clostridium_XlVa*
Otu00045	2.81	0.00	2.89	0.00	2.96	3.20	*Firmicutes*	*Lachnospiraceae*	*Lachnospiracea_incertae_sedis*
Otu00023	3.07	2.74	2.80	0.00	0.00	2.76	*Firmicutes*	*Lachnospiraceae*	*Anaerostipes*
Otu00164	2.52	2.02	2.07	2.05	2.17	0.00	*Firmicutes*	*Ruminococcaceae*	uncl. from *Ruminococcaceae*
Otu00071	0.00	2.48	2.41	2.44	0.00	2.66	*Proteobacteria*	*Enterobacteriaceae*	*Citrobacter*
Otu00063	0.00	2.32	2.35	0.00	2.65	2.65	*Firmicutes*	*Erysipelotrichaceae*	*Erysipelotrichaceae_incertae_sedis*
Otu00002	0.00	0.00	0.00	0.00	3.83	4.20	*Bacteroidetes*	*Bacteroidaceae*	*Bacteroides*
Otu00011	3.73	3.42	3.36	3.42	-3.49	-3.24	*Bacteroidetes*	*Bacteroidaceae*	*Bacteroides*
Otu00141	2.50	0.00	2.00	0.00	2.09	0.00	*Firmicutes*	*Ruminococcaceae*	uncl. from *Ruminococcaceae*
Otu00051	0.00	2.56	0.00	0.00	2.62	0.00	*Firmicutes*	*Erysipelotrichaceae*	*Clostridium_XVIII*
Otu00056	0.00	2.10	2.02	0.00	0.00	0.00	*Firmicutes*	*Lachnospiraceae*	*Blautia*
Otu00028	0.00	0.00	0.00	0.00	0.00	2.93	*Bacteroidetes*	*Porphyromonadaceae*	*Parabacteroides*
Otu00175	2.51	0.00	0.00	0.00	0.00	0.00	*Proteobacteria*	*Desulfovibrionaceae*	*Bilophila*
Otu00041	2.66	0.00	0.00	0.00	2.79	-3.40	*Firmicutes*	*Clostridiaceae*	*Clostridium_sensu_stricto*
Otu00078	-2.79	0.00	0.00	0.00	2.24	2.17	*Firmicutes*	*Ruminococcaceae*	uncl. from *Ruminococcaceae*
Otu00035	0.00	-2.66	0.00	0.00	0.00	2.87	*Firmicutes*	*Lachnospiraceae*	*Blautia*
Otu00067	2.44	0.00	0.00	0.00	0.00	-2.26	*Firmicutes*	*Ruminococcaceae*	*Butyricicoccus*
Otu00113	0.00	0.00	0.00	0.00	-2.04	0.00	*Firmicutes*	unclassified	unclassified
Otu00204	-2.13	0.00	0.00	0.00	0.00	0.00	*Firmicutes*	*Lachnospiraceae*	uncl. from *Lachnospiraceae*
Otu00037	0.00	0.00	0.00	0.00	-2.18	0.00	*Firmicutes*	*Lachnospiraceae*	uncl. from *Lachnospiraceae*
Otu00182	-2.19	0.00	0.00	0.00	0.00	0.00	*Firmicutes*	*Lachnospiraceae*	uncl. from *Lachnospiraceae*
Otu00207	-2.29	0.00	0.00	0.00	0.00	0.00	*Firmicutes*	*Ruminococcaceae*	*Clostridium_IV*
Otu00102	-2.34	0.00	0.00	0.00	0.00	0.00	*unclassified*	*unclassified*	*unclassified*
Otu00149	-2.37	0.00	0.00	0.00	0.00	0.00	*Firmicutes*	*Ruminococcaceae*	uncl. from *Ruminococcaceae*
Otu00076	-2.42	0.00	0.00	0.00	0.00	0.00	*Bacteroidetes*	*Prevotellaceae*	*Prevotella*
Otu00007	0.00	0.00	0.00	-3.26	-3.23	3.47	*Bacteroidetes*	*Bacteroidaceae*	*Bacteroides*
Otu00022	0.00	0.00	0.00	0.00	0.00	-3.82	*Firmicutes*	*Lachnospiraceae*	*Coprococcus*
Otu00162	-2.25	0.00	0.00	0.00	-2.05	0.00	*Firmicutes*	*Lachnospiraceae*	uncl. from *Lachnospiraceae*
Otu00199	-2.22	0.00	0.00	0.00	-2.10	0.00	*Actinobacteria*	*Coriobacteriaceae*	*Slackia*
Otu00062	-2.39	0.00	0.00	0.00	-2.20	0.00	*Proteobacteria*	*Hyphomicrobiaceae*	*Gemmiger*
Otu00080	-2.36	0.00	0.00	0.00	0.00	-2.59	*Actinobacteria*	*Coriobacteriaceae*	*Slackia*
Otu00048	-2.69	0.00	0.00	-2.61	-2.59	2.52	*Firmicutes*	*Lachnospiraceae*	*Roseburia*
Otu00024	-3.08	-3.35	0.00	0.00	0.00	0.00	*Actinobacteria*	*Coriobacteriaceae*	*Eggerthella*
Otu00136	-2.56	-2.20	0.00	0.00	-2.37	0.00	*Firmicutes*	*Clostridiales_Incertae_Sedis_XI*	*Finegoldia*
Otu00016	0.00	-3.92	0.00	0.00	-3.88	0.00	*Firmicutes*	*Peptostreptococcaceae*	*Peptostreptococcus*
Otu00077	-2.22	-2.08	-2.02	-2.07	0.00	0.00	*Firmicutes*	*Lachnospiraceae*	*Lachnospiracea_incertae_sedis*
Otu00032	0.00	0.00	-2.87	0.00	-3.07	-2.81	*Firmicutes*	*Lachnospiraceae*	*Clostridium_XlVa*
Otu00017	-3.59	-3.43	-3.41	-3.47	0.00	2.88	*Bacteroidetes*	*Porphyromonadaceae*	*Parabacteroides*
Otu00053	-2.57	-2.41	-2.21	-2.36	0.00	-2.05	*Firmicutes*	*Lachnospiraceae*	*Blautia*
Otu00050	0.00	-2.79	-2.77	0.00	-3.05	-3.10	*Firmicutes*	*Lachnospiraceae*	*Clostridium_XlVa*
Otu00114	-2.61	-2.36	-2.19	-2.24	-2.41	0.00	*Firmicutes*	*Ruminococcaceae*	uncl. from *Ruminococcaceae*
Otu00060	-2.35	-2.31	0.00	-2.48	-2.51	-2.30	*Firmicutes*	*Lachnospiraceae*	*Lachnospiracea_incertae_sedis*
Otu00015	3.38	-3.64	-3.44	0.00	-4.19	-4.65	*Firmicutes*	*Clostridiaceae*	*Clostridium_sensu_stricto*
Otu00036	-2.95	-2.80	-2.47	-2.89	0.00	-2.32	*Actinobacteria*	*Bifidobacteriaceae*	*Bifidobacterium*
Otu00079	-2.45	-2.20	-2.22	-2.37	-2.47	-2.22	*Firmicutes*	*Ruminococcaceae*	uncl. from *Ruminococcaceae*
Otu00020	-3.81	-3.38	-3.42	-3.63	-3.40	3.51	*Proteobacteria*	*Sutterellaceae*	*Parasutterella*
Otu00124	-2.64	-2.49	-2.35	-2.24	-2.27	-2.30	*Firmicutes*	*Clostridiaceae*	*Clostridium_sensu_stricto*
Otu00091	-2.41	-2.46	-2.47	-2.19	-2.44	-2.39	*Firmicutes*	*Lachnospiraceae*	uncl. from *Lachnospiraceae*
Otu00049	-2.74	-2.59	-2.64	-2.58	-2.53	-2.21	*Firmicutes*	*Lachnospiraceae*	uncl. from *Lachnospiraceae*
Otu00003	-4.14	-4.02	-3.99	-3.99	-3.81	3.86	*Firmicutes*	*Ruminococcaceae*	*Flavonifractor*
Otu00087	-3.16	-2.82	-2.76	-2.72	-2.66	-2.15	*Firmicutes*	*Ruminococcaceae*	uncl. from *Ruminococcaceae*
Otu00064	-2.59	-2.58	-2.76	-2.20	-3.07	-3.36	*Firmicutes*	*Lachnospiraceae*	uncl. from *Lachnospiraceae*

Supplementary Table 2: Continued.

Otu00025	-3.61	-3.37	-3.42	-3.36	-3.38	0.00	*Firmicutes*	*Veillonellaceae*	*Dialister*
Otu00005	-3.65	-3.36	-3.46	-3.48	-3.68	0.00	*Bacteroidetes*	*Bacteroidaceae*	*Bacteroides*
Otu00033	-3.37	-3.23	-3.24	-3.39	-3.28	-3.14	*Firmicutes*	*Peptostreptococcaceae*	*Clostridium_XI*
Otu00027	-3.31	-3.52	-3.32	-3.41	-3.34	-3.20	*Firmicutes*	*Ruminococcaceae*	*Flavonifractor*
Otu00039	-3.81	-3.73	-3.62	-3.78	-3.55	-2.15	*Firmicutes*	*Clostridiales_Incertae_ Sedis_XI*	*Peptoniphilus*
Otu00038	-2.87	-3.70	-3.68	-3.78	-3.53	-3.21	*Firmicutes*	*Ruminococcaceae*	*Oscillibacter*
Otu00001	-4.32	-4.38	-4.38	-4.44	-4.11	0.00	*Bacteroidetes*	*Bacteroidaceae*	*Bacteroides*
Otu00004	-4.36	-4.37	-4.39	-4.30	-4.34	0.00	*Firmicutes*	*Lachnospiraceae*	*Clostridium_XlVa*
Otu00026	-3.63	-3.64	-3.74	-3.64	-3.75	-4.14	*Firmicutes*	*Lachnospiraceae*	*Dorea*
Otu00014	-4.05	-4.02	-4.02	-4.00	-4.02	-3.86	*Actinobacteria*	*Coriobacteriaceae*	*Collinsella*

OTUs with negative LDA scores (red) are enriched in samples of children fecal microbiota cultured in *C. difficile* conditioned media, while OTUs with positive LDA scores (blue) are enriched in samples of children fecal microbiota only. OTUs are listed according to LDA score in a descending manner.

uncl., unclassified; WUniFrac, weighted UniFrac; E27 and 1998, ribotype 027 strains; 3014 and 1974, ribotype 176 strains; W43018 and 4894, ribotype 078 strains

Supplementary Table 3: Comparison of significantly increased OTUs in control samples (children fecal microbiota only) and in samples of co-cultures children fecal microbiota/*C. difficile* ribotypes 027, 176 and 078 strains.

combination of fecal microbiota and *C. difficile* ribotype	increased in co-culture *C. difficile*/microbiota	increased in control (microbiota only)
children microbiota+027 (E27)	*Escherichia_Shigella* (Otu00008)	uncl. from *Lachnospiraceae* (Otu00012)
	Clostridium_XIVa (Otu00004)	*Peptostreptococcus* (Otu00016)
	Morganella (Otu00006)	*Flavonifractor* (Otu00003)
	Bacteroides (Otu00005)	*Coprococcus* (Otu00022)
	Bacteroides (Otu00007)	Streptococcus (Otu00029)
children microbiota+027 (1998)	*Clostridium_sensu_stricto* (Otu00015)	*Clostridium_XlVb* (Otu00019)
	Bacteroides (Otu00001)	*Flavonifractor* (Otu00003)
	Clostridium_XIVa (Otu00004)	*Peptostreptococcus* (Otu00016)
	Escherichia_Shigella (Otu00008)	*Veillonella* (Otu00013)
	Bacteroides (Otu00011)	*Coprococcus* (Otu00022)
children microbiota+176 (3014)	*Escherichia_Shigella* (Otu00008)	uncl. from *Lachnospiraceae* (Otu00012)
	Bacteroides (Otu00001)	*Flavonifractor* (Otu00003)
	Clostridium_XIVa (Otu00004)	*Peptostreptococcus* (Otu00016)
	Morganella (Otu00006)	*Veillonella* (Otu00013)
	Clostridium_sensu_stricto (Otu00015)	*Coprococcus* (Otu00022)
children microbiota+176 (1974)	*Clostridium_sensu_stricto* (Otu00015)	*Flavonifractor* (Otu00003)
	Escherichia_Shigella (Otu00008)	*Peptostreptococcus* (Otu00016)
	Bacteroides (Otu00001)	*Veillonella* (Otu00013)
	Bacteroides (Otu00005)	*Coprococcus* (Otu00022)
	Bacteroides (Otu00011)	*Morganella* (Otu00006)
children microbiota+078 (W43018)	*Clostridium_XIVa* (Otu00004)	uncl. from *Lachnospiraceae* (Otu00012)
	Morganella (Otu00006)	*Peptostreptococcus* (Otu00016)
	Bacteroides (Otu00001)	*Flavonifractor* (Otu00003)
	Escherichia_Shigella (Otu00008)	*Coprococcus* (Otu00022)
	Bacteroides (Otu00005)	*Veillonella* (Otu00013)
children microbiota+078 (4894)	*Morganella* (Otu00006)	*Peptostreptococcus* (Otu00016)
	Bacteroides (Otu00001)	*Veillonella* (Otu00013)
	Enterococcus (Otu00021)	*Flavonifractor* (Otu00003)
	Bacteroides (Otu00007)	*Coprococcus* (Otu00022)
	Bacteroides (Otu00011)	*Oscillibacter* (Otu00043)

Presented OTUs were identified by the LEfSe test (mothur software), which uses linear discriminant analysis (LDA) to find OTUs that significantly differ in abundance between cultures of microbiota only (control) and co-cultures of microbiota and *C. difficile*. For each comparison only top 5 OTUs with highest LDA scores are presented. For additional information see S1 Table.
uncl., unclassified; E27 and 1998, ribotype 027 strains; 3014 and 1974, ribotype 176 strains; W43018 and 4894, ribotype 078 strains

Supplementary Table 4: Comparison of significantly increased OTUs in control samples (children fecal microbiota only) and in samples of children fecal microbiota cultured in conditioned media of *C. difficile* ribotypes 027, 176 and 078 strains.

combination of fecal microbiota and *C. difficile* ribotype	increased in *C. difficile* conditioned medium	increased in control (microbiota only)
children microbiota+027 (E27)	*Clostridium_XIVa* (Otu00004)	uncl. from *Lachnospiraceae* (Otu00012)
	Bacteroides (Otu00001)	*Morganella* (Otu00006)
	Flavonifractor (Otu00003)	*Veillonella* (Otu00013)
	Collinsella (Otu00014)	*Clostridium_XVIII* (Otu00010)
	Parasutterella (Otu00020)	*Enterococcus* (Otu00021)
children microbiota+027 (1998)	*Bacteroides* (Otu00001)	uncl. from *Lachnospiraceae* (Otu00012)
	Clostridium_XIVa (Otu00004)	*Veillonella* (Otu00013)
	Flavonifractor (Otu00003)	*Morganella* (Otu00006)
	Collinsella (Otu00014)	*Clostridium_XVIII* (Otu00010)
	Peptostreptococcus (Otu00016)	*Enterococcus* (Otu00021)
children microbiota+176 (3014)	*Clostridium_XIVa* (Otu00004)	uncl. from *Lachnospiraceae* (Otu00012)
	Bacteroides (Otu00001)	*Veillonella* (Otu00013)
	Collinsella (Otu00014)	*Morganella* (Otu00006)
	Flavonifractor (Otu00003)	*Clostridium_XVIII* (Otu00010)
-	*Dorea* (Otu00026)	*Enterococcus* (Otu00021)
children microbiota+176 (1974)	*Bacteroides* (Otu00001)	uncl. from *Lachnospiraceae* (Otu00012)
	Clostridium_XIVa (Otu00004)	*Veillonella* (Otu00013)
	Collinsella (Otu00014)	*Morganella* (Otu00006)
	Flavonifractor (Otu00003)	*Enterococcus* (Otu00021)
	Peptoniphilus (Otu00039)	*Clostridium_XVIII* (Otu00010)
children microbiota+078 (W43018)	*Clostridium_XIVa* (Otu00004)	uncl. from *Lachnospiraceae* (Otu00012)
	Clostridium_sensu_stricto (Otu00015)	*Morganella* (Otu00006)
	Bacteroides (Otu00001)	*Veillonella* (Otu00013)
	Collinsella (Otu00014)	*Clostridium_XVIII* (Otu00010)
	Peptostreptococcus (Otu00016)	*Enterococcus* (Otu00021)
children microbiota+078 (4894)	*Clostridium_sensu_stricto* (Otu00015)	*Morganella* (Otu00006)
	uncl. from *Lachnospiraceae* (Otu00012)	*Veillonella* (Otu00013)
	Dorea (Otu00026)	*Bacteroides* (Otu00002)
	Collinsella (Otu00014)	*Clostridium_XVIII* (Otu00010)
	Coprococcus (Otu00022)	*Enterococcus* (Otu00021)

Presented OTUs were identified by the LEfSe test (mothur software), which uses linear discriminant analysis (LDA) to find OTUs that significantly differ in abundance between cultures of microbiota only (control) and cultures of microbiota in *C. difficile* conditioned media. For each comparison only top 5 OTUs with highest LDA scores are presented. For additional information see S2 Table.

uncl., unclassified; E27 and 1998, ribotype 027 strains; 3014 and 1974, ribotype 176 strains; W43018 and 4894, ribotype 078 strains
